# Ground Squirrel Shooting and Potential Lead Exposure in Breeding Avian Scavengers

**DOI:** 10.1371/journal.pone.0167926

**Published:** 2016-12-12

**Authors:** Garth Herring, Collin A. Eagles-Smith, Mason T. Wagner

**Affiliations:** 1 United States Geological Survey, Forest and Rangeland Ecosystem Science Center, Corvallis, OR, United States of America; 2 Oregon State University, Department of Fisheries and Wildlife, Corvallis, OR, United States of America; University of Lleida, SPAIN

## Abstract

Recreational ground squirrel shooting is a popular activity throughout the western United States and serves as a tool for managing ground squirrel populations in agricultural regions. Belding’s ground squirrels (*Spermophilus beldingi*) are routinely shot in California, Nevada, and Oregon across habitats that overlap with breeding avian scavengers. Ground squirrels shot with lead (Pb)-based bullets may pose a risk to avian scavengers if they consume carcasses containing Pb fragments. To assess the potential risk to breeding avian scavengers we developed a model to estimate the number, mass, and distribution of Pb fragments in shot ground squirrels using radiographic images. Eighty percent of shot carcasses contained detectible Pb fragments with an average of 38.6 mg of Pb fragments. Seven percent of all carcasses contained Pb fragment masses exceeding a lethal dose for a model raptor nestling (e.g. American kestrel *Falco sparverius*). Bullet type did not influence the number of fragments in shot ground squirrels, but did influence the mass of fragments retained. Belding’s ground squirrels shot with .17 Super Mag and unknown ammunition types contained over 28 and 17 times more mass of Pb fragments than those shot with .22 solid and .22 hollow point bullets, respectively. Ground squirrel body mass was positively correlated with both the number and mass of Pb fragments in carcasses, increasing on average by 76% and 56% respectively across the range of carcass masses. Although the mass of Pb retained in ground squirrel carcasses was small relative to the original bullet mass, avian scavenger nestlings that frequently consume shot ground squirrels may be at risk for Pb-induced effects (e.g., physiology, growth, or survival). Using modeling efforts we found that if nestling golden eagles (*Aquila chrysaetos*), red-tailed hawks (*Buteo jamaicensis*), and Swainson’s hawks (*B*. *swainsoni*) consumed shot ground squirrels proportionately to the nestling’s mass, energy needs, and diet, 100% of the nestling period would exceed a 50% reduction in delta-aminolevulinic acid dehydratase production threshold, the last 13–27% of the nestling stage would exceed a reduced growth rate threshold, but no nestlings would be expected to exceed a level of Pb ingestion that would be lethal.

## Introduction

Lead (Pb) exposure is a serious conservation threat to birds [1] because it causes significant behavioral and physiological impairment as well as direct mortality at environmentally-relevant exposures [1–4]. Although avian Pb exposure has been associated with numerous sources such as Pb-based paints [5], mining [6,7], and fishing tackle [8,9], spent ammunition is often the most widespread source of Pb to scavenging birds [10–16]. Carcasses from big game hunting are a well-described vector of Pb exposure to avian scavengers [14,17,18], but other shooting and hunting activities may also cause Pb exposure in avian scavengers.

Recreational varmint shooting may provide a substantial delivery vehicle for Pb exposure to avian scavengers [19–21], especially in regions of the western United States where ground squirrel and prairie dog populations (hereafter ground squirrel, e.g., Richardson’s ground squirrel *Urocitellus richardsonii*; [20], black-tailed prairie dogs *Cynomys ludovicianus*; [21]) overlap with agriculture or ranching activities and are considered major pests. Damage to agricultural crops (e.g., alfalfa *Medicago sativa*) across the central and western United States from mammalian pests can result in substantial financial loss, thus shooting may be a valuable and effective management tool [22,23]. Ground squirrel shooting is generally unregulated (although in some states a hunting license is required), and shot ground squirrel carcasses are commonly left in fields where they can be consumed by scavengers. The extremely high densities of ground squirrels in some areas (up to 300 ha^-1^; [22]), make it possible for a single person to shoot hundreds of squirrels per day from a single prairie dog colony or agricultural field [19]. The total number of ground squirrels shot per year across the central and western United States is unknown, but more than 1.1 million ground squirrels were shot in South Dakota in one year alone [24]. Moreover, the expandable Pb bullets that are commonly used for this activity may distribute Pb fragments throughout ground squirrel carcass [21], potentially enhancing exposure likelihood for scavenging birds. In North America there are at least 13 bird species that commonly scavenge dead ground squirrels, including bald eagles (*Haliaeetus leucocephalus*), common ravens (*Corvus corax*), ferruginous hawks (*Buteo regalis*), golden eagles (*Aquila chrysaetos*), red-tailed hawks (*Buteo jamaicensis*), and Swainson's hawks (*B*. *swainsoni*) [25–27].

Little is quantitatively known about the extent to which shot ground squirrels may act as a vector for Pb to avian scavengers. More specifically, an understanding of the number of bullet fragments, their mass, and distribution within shot ground squirrels carcasses is critical for understanding risk to avian scavengers, particularly during their breeding season when ground squirrels may comprise a substantial portion of nestling diets; see [28–30]. The number and mass of Pb fragments remaining in a carcass is important because many small Pb fragments in a carcass allow for Pb to be more easily ingested [31,32]. Additionally, smaller size fragments increase the effective surface area of Pb in the intestinal tract, which allows Pb to be more readily absorbed into the blood stream by scavengers [33]. Further if small fragments are distributed throughout much of a shot ground squirrel carcass, avian scavengers may be more likely to consume those fragments in comparison to either larger fragments or an unfragmented bullet which can be more easily avoided or regurgitated [31,32]. Finally, there is a range of bullet types and calibers commonly used to shoot ground squirrels. Differences in bullet fragmentation patterns associated with bullet types and calibers may influence the number, mass, and distribution of fragments in shot ground squirrels [34].

Our objectives in this study were to quantify the potential for shot grounds squirrels to act as a vector for Pb exposure in breeding avian scavengers and determine if specific types of commonly used ammunition differed with respect to numbers, mass, or distribution of fragments in carcasses. To do so, we examined shot Belding’s ground squirrels (*U*. *beldingi*) from California and Oregon, and quantified the number, mass, and distribution of fragments in carcasses relative to ammunition types using radiography. We validated radiography results by extracting bullet fragments from a subset of shot ground squirrel carcasses. Additionally, using estimates of Pb fragments from shot ground squirrels, we modeled potential Pb exposure in nestling raptors relative to published toxicity benchmarks (physiology, growth, and survival) while accounting for nestling age, diet, food mass requirements, and Pb content of shot ground squirrels. We discuss these results in the context of potential exposure to breeding avian scavengers that routinely scavenge at shooting fields and bring ground squirrels to their nestlings.

## Methods

### Ethics Statement

We made every attempt to reduce disturbance, stress, and other impacts to target and non-target species during the course of this research. We conducted the research and salvaged dead ground squirrels under Oregon Department of Fish and Wildlife Research Permits 009–14 and 064–15.

### Study area

The western United States (US) are major producers of alfalfa in the US, with approximately 40% of the US production and nearly 3 million ha of alfalfa acreage coming from just 11 western states [35]. Within California, Nevada and Oregon, alfalfa is one of the top three crops in acreage [35], and yield losses due to ground squirrel damage can be as high as 18–46% in some areas [22]. Ground squirrel densities in alfalfa fields can vary considerably and have been reported to range from 12–296 ha^-1^ [22]. We conducted our research in Lake (43.228628° N -120.939178° W) and Malheur Counties (43.406875° N -118.715316° W) OR and Siskiyou County (41.823356° N -121.700228° W) CA USA, three agricultural regions with extensive alfalfa production, widespread ground squirrel shooting activity, and wide-ranging breeding scavenging bird populations including Golden Eagles.

During 2014 and 2015, we collected carcasses of shot ground squirrels from these three areas. Ground squirrel shooting routinely occurs throughout all three areas, both as a recreational activity and as a tool to manage ground squirrel populations. These activities mostly occur between March, when ground squirrels emerge from hibernation, and August, when ground squirrels often begin hibernation.

### Ground squirrel sampling

We salvaged shot ground squirrels immediately after shooting events on alfalfa fields under two different scenarios; 1) squirrels that were shot with mixed, unknown ammunition types (*n* = 6 fields), and 2) squirrels that were shot with known ammunition types (*n* = 5 fields). In the case ground squirrels shot with known ammunition types, details on ammunition type were provided by land owners or private individuals that shot ground squirrels. The first approach to ground squirrel carcass collections allowed us to assess the number of fragments, distribution, and mass of fragments from carcasses that were readily available on the landscape. Secondly, the collection of carcasses shot with known ammunition type allowed us to determine if specific types of ammunition resulted in different numbers, distribution, or mass of Pb fragments in shot carcasses. All ground squirrels were shot and killed under natural field conditions at distances ranging from approximately 20–150 m. In order to ensure that Pb fragments in salvaged ground squirrels were representative of common conditions, ground squirrels were shot and retrieved by private citizens engaged in this activity, or by private land-owners that shot, retrieved, and then donated carcasses. No ground squirrels were shot specifically for the purposes of this study and U.S. Geological Survey staff did not shoot any of the ground squirrels in the this study. Specimens were placed in polyethylene storage bags, labeled with ammunition type, placed on wet ice in the field, and then stored frozen at -20°C until processing and analysis.

### Laboratory methods

In the laboratory, we weighed carcasses to the nearest 0.01 g using Ohaus Adventure Pro balance (Ohaus Corporation, Parsippany, New Jersey, USA), removed the dermal tissue (skin and fur) from each specimen, and stored them frozen in plastic bags until radiographic analysis. The dermal tissue was removed from each carcass to facilitate improved digestion and extraction of bullet fragments from carcasses. We assumed that the dermal layer retained a limited amount of Pb and is not a significant source of Pb to avian scavengers because it is typically regurgitated upon consumption [36,37]. However, we radiographed each pelage to validate this assumption. We radiographed all carcasses and pelage samples at the Oregon Zoo Veterinary Medical Center using a Del Medical Linear MC-150 radiograph gantry (UMG/DEL Medical, Harrison New York, USA) equipped with a Del Medical CM Series control panel. For all radiographs, we set amperage to 2.0 mAs and voltage to 50 kVp. We positioned carcasses ventral side up next to their dermal tissue and with a scale of known size.

**Fig 1:**
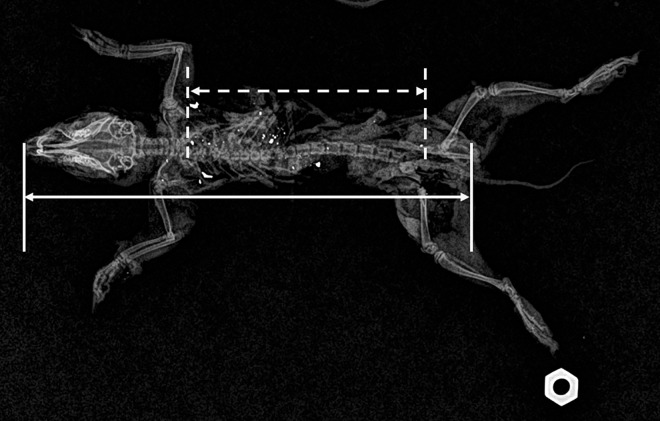
Radiograph of Belding’s ground squirrel carcass shot with a 20 grain .17 Super Mag bullet. Bullet fragments appear as white flecks throughout the carcass. Total number of fragments recovered from the carcass was 156; 97% (n = 151) consisted strictly of Pb with a total mass of 61 mg of Pb. Dashed horizontal and vertical lines illustrate how the maximum distribution of bullet fragments was measured relative to the length of carcass (base of the tail to most distal portion of the head) illustrated by the solid lines. White scale object in lower right of image is 12.6 mm in width.

We analyzed all radiograph images using ImageJ software (J 1.46r; [38]), and converted them to an 8-bit grayscale images. Individual pixels were automatically assigned an exposure value given their resistance to the radio waves (e.g., metal fragments completely resist radio waves). Using the threshold adjustment option, we converted each digital radiograph into a binary file, creating a black and white image based upon the previously assigned exposure values. We manually adjusted the threshold settings to 130; pixels above this value were converted to white, and pixels below the value were converted to black [38]. Once all fragments were identified, we recorded the number of fragments, number of pixels in each fragment, and the total combined number per ground squirrel. Pixels were converted to an area estimate (mm^2^) by calibrating the digital image using a scale of known size that was next to each carcass during radiograph acquisition (Fig 1). We measured the distribution of fragments throughout carcasses by first measuring the maximum distance observed between bullet fragments as well as the length of the carcass from the base of the tail to most distal portion of the head (Fig 1). We then calculated the distribution of fragments as a ratio of the distance between the two farthest apart fragments relative to the length of the carcass.

In order to validate the accuracy of radiograph images for estimating bullet fragment numbers, distribution, and mass in ground squirrels, we extracted bullet fragments from a subset of carcasses (*n* = 30) using a digestion procedure. We used a three-step process to separate bullet fragments from each carcass. (1) We conducted a preliminary tissue decomposition step in a pressure cooker (Maxi-Matic; City of Industry, California, USA) with 1 liter of deionized water for 9 hours at a maximum pressure of 90 kpa and temperature of 120°C. (2) We then further digested the squirrels in a 2.5% solution of WME-2717, an industrial enzyme blend, (Creative Biomart; Shirley, New York, USA), used to treat drainage systems at slaughterhouses by rapidly digesting protein. We digested each squirrel in solution at 50°C for 7–10 days. To stop the enzyme digestion process, we added a 10% bleach solution. We then stored the resulting solution in a refrigerator at 4C until further processing. (3) To extract bullet fragments from the digest, we first filtered the solution through 500μm and 210μm sieves to remove bones and large, undigested tissue particles. Sieves were examined under a dissecting microscope to ensure no visible bullet fragments remained. We then used a gold prospecting sluice box (Back Packer Sluice, Angus MacKirk; Garden City, Idaho, USA) to recover bullet fragments from the remaining carcass solution. We positioned the sluice box at an angle of 12° with running water flowing at variable rates, and then gradually fed material from the digest into the sluice to ensure maximum capture of bullet particles. All material that passed through the sluice box was collected in a container at the end of the sluice box. Bullet fragment material caught in the sluice box riffles were extracted with forceps or by using a 5 mL bulb pipette and placed in labeled 25 mL glass scintillation vials. All carcass material was processed twice through the sluice box. Material collected from the sluice box riffles was then oven dried at 50°C in uncapped scintillation vials. Once dry, vials were capped and stored at room temperature. We visually inspected all fragments to determine if they were Pb core material, jacket material, or combined Pb/jacket fragment material under a stereomicroscope (model M3Z, Wild Heerbrugg; Heerbrugg, Switzerland), separating the three fragment types. Recovered fragments were individually weighed using a micro balance (± 0.001mg; model Pro 11, Satorius; Bohomeia, New York, USA). Fragments that were too small to be weighed on their own because they could not be handled without breaking were counted and weighed together as an aggregate.

#### Laboratory methods validation

In order to test whether the digest and extraction procedure resulted in any appreciable digestion and loss of solid Pb particles, we subjected Pb fragments spanning a range of sizes (1.3–154 mg, *n* = 78) to the same procedures, and then measured fragment masses pre and post tissue decomposition. Pb fragments subjected to this procedure lost an average 1.6% (± 0.2) of their initial mass indicating there was a minimal effect from this procedure. We also tested whether the enzyme solution would change the mass of Pb fragments by placing 15 Pb fragments of varying mass (3–28.6 mg) in the same solution and following the identical procedures as above. As with the initial decomposition step, the enzyme solution did not have an appreciable influence on the mass of Pb fragments (mean mass loss = 0.42% ± 0.18). Finally, we tested the efficacy of fragment capture through the sluice box by adding 10 Pb bullet fragments of varying masses (6–35 mg) each to the digests of two separate ground squirrel carcasses that had not been shot but were salvaged after being hit by a vehicle on an adjacent highway to the study area. The sluice box operator was blind to the number of fragments. In both trials, 100% of the fragments were recovered using the gold sluice box procedure.

### Statistical methods

To estimate the number of bullet fragments in shot ground squirrel carcasses, we used a two-step statistical procedure. We first developed a linear regression model to estimate the number of fragments in shot carcasses using the radiograph estimate of fragments. Using a subset of the carcasses where we had both radiograph fragment counts and digestion fragments counts, our linear regression model included the number of digested fragments as the response variable and the radiograph count as the independent variable. We used the parameter estimates from this linear regression model to estimate the number of fragments across all ground squirrel carcasses (mixed unknown or known caliber and bullet type). This approach allowed us to estimate bullet fragment numbers in carcasses that were only radiographed, and not digested. We excluded data from one ground squirrel carcass from all analyses because the bullet fragments in the radiograph were so highly clustered in the head region of the ground squirrel that they could not be counted or measured accurately by the Image J software. In this case, the Image J software identified 94 fragments in the carcass, but the extracted carcass actually contained 398 bullet fragments. Removing this data point decreased the value of the beta coefficient estimate, thus our estimates derived from this model were lower than if we had left this outlier in the model. We then tested whether the number of estimated fragments in carcasses differed by type of ammunition (e.g., mixed unknown or known caliber and bullet type) using an analysis of covariance (ANCOVA) model. The estimated number of fragments was included as the response variable, with type of ammunition as the independent variable. We included ground squirrel mass as a covariate to control for variation in squirrel size differences because individuals ranged in mass from 54–521 g during shooting activities. We also included an interaction term for ammunition type × ground squirrel mass in order to test for slope differences among bullet types across the mass of shot ground squirrels. We tested for differences in the distribution of bullet fragments throughout shot ground squirrel carcasses by the type of ammunition (mixed unknown or known caliber and bullet type) using an ANCOVA model. We included the percent distribution of fragments as the response variable, bullet type as the independent variable, carcass mass as a covariate to control for size differences among shot ground squirrel carcasses, and an interaction term for ammunition type × ground squirrel mass.

Similar to estimating the number of bullet fragments in shot ground squirrel carcasses above, we used a two-step statistical procedure to estimate the total mass (mg) of bullet fragments in shot ground squirrel carcasses. We first developed a linear regression model to estimate the total mass of bullet fragments in shot carcasses using the radiograph estimate of fragment area (mm^2^; 21). Using a subset of the carcasses where we had both fragment area (from radiographs) and bullet fragment mass (from digests), our linear regression model included fragment mass as the response variable and fragment area as the independent variable. We used the parameter estimates from this linear regression model to estimate the total mass of fragments across all ground squirrel carcasses (mixed unknown or known caliber and bullet type). We then tested whether the estimated mass of Pb fragments in carcasses differed by ammunition type (e.g., mixed unknown or known caliber and bullet type) using an ANCOVA model where the estimated mass of fragments was the response variable, and ammunition type was the independent variable. We included mass of ground squirrel carcasses as a covariate to control for carcass size differences and an interaction term for ammunition type × ground squirrel mass. Lastly, to determine if there was a relationship between the number of bullet fragments and mass of bullet fragments in shot ground squirrel carcasses we ran a correlation analysis where the log estimated fragment mass was the response variable and log estimated number of bullet fragments was the independent variable. We dropped interaction terms from all final models if they were non-significant (*P*>0.10) and natural log-transformed data to improve normality of residuals where appropriate.

After validating the approaches described above to estimate the number and mass of Pb fragments retained in ground squirrels after being shot, we developed several modeling scenarios for Pb exposure in nestlings of three bird species that regularly consume scavenged ground squirrels (golden eagle, red-tailed hawk, and Swainson’s hawk). We calculated estimates of daily ground squirrel consumption rates (g/day) for each species based upon nestling growth, food requirements, and diet selection. We then applied our estimates of Pb availability in shot ground squirrels (mg Pb/g ground squirrel) to model potential daily Pb exposure associated with the ground squirrel consumption for each species. Finally we applied those estimates to thresholds for toxicity effects (physiology, growth, and survival) based on published data.

Nestling growth rates (mass) were adjusted for each day in the nest based upon growth models derived from nest observations for each species [39–42]. The proportion of golden eagle, red-tailed hawk, and Swainson’s hawk diet derived from ground squirrels was assigned for each species (29%, 42%, 40%, respectively) based upon published studies in states where ground shooting is common and overlaps with nesting of each bird species [28,29,43]. Dietary Pb-content was assigned a value of 0.21 mg Pb/g of ground squirrel, the mean value from estimates of Pb fragments retained in ground squirrel carcasses.

A threshold for physiological effects was estimated from a Pb dosing study on red-tailed hawks. Red-tailed hawk adults dosed with 0.82 mg Pb/kg body weight suffered a 55% reduction in delta-aminolevulinic acid dehydratase activity after seven days exposure; cumulative consumption was approximately 6.34 mg of Pb or 5.74 mg/kg body mass over that time period [44]. Lead can impair the production of delta-aminolevulinic acid dehydratase, a precursor for heme synthesis [1,6,12]. Elevated Pb concentrations resulting in decreased heme synthesis can cause anemia in birds [1]. A threshold for growth effects was estimated from a Pb dosing study on American kestrels (*Falco sparverius*). Growth rates were impaired by Pb at a dosing rate of 125 mg Pb/kg body mass, resulting in lower growth rates than controls after 4 days of exposure and a cumulative consumption of approximately 6 mg of Pb or 306 mg/kg body mass [45]. A survival threshold was estimated using the same study [45]. Forty percent of dosed (625 mg Pb/kg body mass) kestrel chicks died after six days of exposure and a cumulative Pb consumption of approximately 70 mg of Pb (range = 60–80 mg of Pb; [45]) or 2294 mg/kg body mass. We calculated cumulative daily exposure (mg Pb/kg body mass) based upon the above criteria (hereafter field conditions model). In doing so, we assumed that 100% of the Pb ingested from shot ground squirrels was assimilated into the raptor nestling, which is reasonable given that 76% of all fragments were very small (<12.5 mg) [21,32]. Previous experimental research has shown that avian scavengers do not avoid ingesting bullet fragments this small when foraging on carcasses [32]. Thus to calculate cumulative daily Pb exposure in raptor nestlings we used the following formula

$$Pb\;\exp\left(\frac{\mathrm{mg}}{d}\right)=\sum^{d}(m_{f}\times gsd)\times Pb)/m_{n}$$

where *d* is a day in a nest, *m*_*f*_ = mass of food/day required in g, *gsd* = proportion of diet derived from ground squirrels, *Pb* = mass of Pb (mg)/g of ground squirrel, and *m*_*n*_ = nestling mass on each day in the nest in kg. We defined the numbers of days in the nest post hatch as 70, 32, and 32 for golden eagles, red-tailed hawks, and Swainson’s hawks respectively.

Our field conditions model did not allow for the generation of confidence intervals around the daily Pb exposure estimate because it was developed using individual data for each day (either a point estimate [e.g., mass] or a mean [e.g., diet]). To provide a credible interval (95% CI) around our estimates of daily cumulative Pb exposure, we used a Monte Carlo simulation [46] to generate additional data associated with variables in our field conditions model based on their mean and standard deviation derived from multiple studies [28,29,43]. We used Program R [46] to simulate 1000 values for each variable listed above assuming each parameter followed a normal distribution. We then utilized those 1000 iterations in the field conditions model to estimate the stand error and develop our 95% CI for the cumulative daily Pb exposure. We did not have estimates of the mean and standard deviation for the mass of chicks at individual time steps, and therefore did not generate additional data for that variable.

Given the large amount of variation in both the mass of Pb fragments in shot ground squirrels and the proportion of raptor nestling diet that is derived from ground squirrels we developed three additional Pb exposure models to provide insight into the varying effects of diet and the amount of Pb in carcasses on Pb exposure and risk to nestling raptors. (1) We estimated the daily cumulative Pb exposure associated with the maximum proportion diet of derived from ground squirrels (47%, 61%, 71%) for golden eagles, red-tailed hawks, and Swainson’s hawks respectively [28,29,43] while maintaining exposure to Pb constant at 0.21 mg Pb/g of ground squirrel (hereafter maximum ground squirrel diet model). (2) We estimated the daily cumulative Pb exposure associated with the maximum mass of Pb observed in a single shot ground squirrel (195 mg or 1.04 mg/g of ground squirrel mass) for golden eagles, red-tailed hawks, and Swainson’s hawks while maintaining the proportion of diet (29–42%) derived from ground squirrels constant (hereafter maximum Pb mass model). (3) We estimated the daily cumulative Pb exposure associated with the maximum proportion of raptor nestling diet derived from ground squirrels and the maximum mass of Pb observed in a single shot ground squirrel as above (hereafter maximum ground squirrel diet and Pb mass model).

## Results

We salvaged 127 Belding’s ground squirrels in 2014 and 2015 that were shot with a combination of known (*n* = 81) and mixed unknown (*n* = 46) ammunition types. Known ammunition consisted of Hornady.17 HMR V-Max (*n* = 22; Hornady Manufacturing Company, Grand Island NE USA), Hornady .17 Mach II V-Max (*n* = 13), Hornady .17 Win Super Mag (*n* = 6), Remington Thunderbolt .22 solid (*n* = 20; Remington Arms Company, LLC, Madison NC USA), and CCI .22 hollow point (*n* = 20; CCI Ammunition, Lewiston ID USA) rounds (Table 1). We detected and recovered bullet fragments in 24 of the 30 (80%) digested carcasses (Fig 1). Among those 24 carcasses that contained bullet fragments, 100% contained Pb fragments, 21% contained combined Pb and jacket material, and 37% contained jacket fragments. Further, 93% of the associated fragment mass in digested carcasses was exclusively Pb fragments, 6% was bullet jacket material, and 1% was combined Pb and bullet jacket material.

**Table 1:** Bullet fragment numbers and mass of Pb fragments associated with known and unknow bullet types used for shooting Belding's ground squirrels in California and Oregon, USA during 2014 and 2015. Mean and standard error (SE) are back-transformed model derived estimates controlling for the mass of ground squirrels being shot.

Bullet Type	*n*	Original bullet mass (mg)	Original bullet grains	Reported muzzle velocity (m/s)	Estimated fragment #s^a^ per ground squirrel	SE	Estimated Mass of Pb (mg) per ground squirrel	SE	% of original bullet mass	% distribution of fragments in carcasses	SE
Unknown	46	-	-	-	22.21	4.89	7.64	3.44	-	41.70	4.51
.17 HMR^a^	21	1102	17	777	16.26	4.72	2.43	1.36	0.22%	30.13	5.80
.17 Mach II^a^	13	1102	17	640	10.93	4.26	0.81	0.63	0.07%	25.54	8.90
.17 Super Mag^a^	6a	1296	20	914	47.59	21.89	12.90	12.00	1.00%	60.98	8.93
.22 solid^b^	20	2592	40	383	13.45	3.77	0.26	0.15	0.01%	29.21	6.70
.22 hollow point^c^	20	2333	36	384	11.39	3.19	0.45	0.26	0.02%	31.48	5.57

^a^ Hornady

^b^Remington

^c^CCI

### Bullet fragments numbers

**Fig 2:**
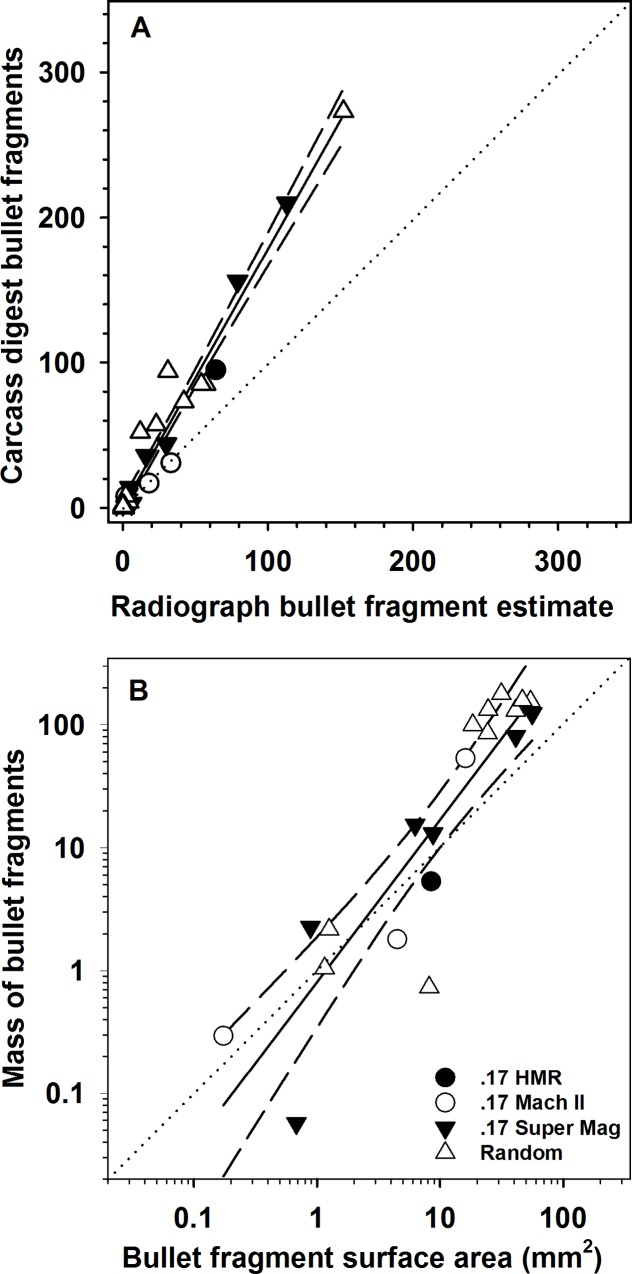
Relationship between (A) number of bullet fragments estimated via radiograph and number of bullet fragments in digested carcass; (B) radiograph-derived estimate of bullet fragment area (mm^2^) and mass of bullet fragments recovered from digested Belding’s ground squirrel carcasses. We used the relationship *y* = 0.870 + 1.774×radiograph bullet fragments numbers to predict numbers of bullet fragments and *y* = -0.217 + 1.321×log total radiograph bullet fragment surface area to predict the log mass of fragments in carcasses we did not digest.

**Fig 3:**
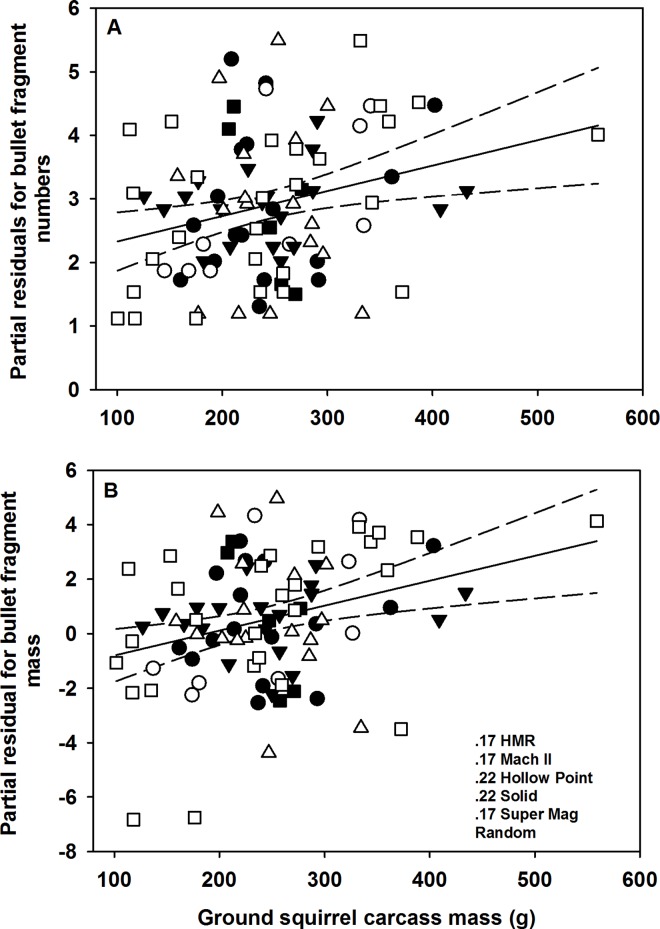
Relationship between numbers of bullet fragments in ground squirrel carcasses (A) and partial residuals for mass of bullet fragments (B) and mass of Belding’s ground squirrel carcasses.

Radiograph-derived estimates of bullet fragment numbers were highly correlated with fragment number recovered from digested ground squirrels (*F*_1,28_ = 26.39, *P* < 0.0001, *R*^2^ = 0.96; Fig 2A). After accounting for the mass of shot ground squirrel carcasses we found no effect of bullet type on the number of bullet fragments in shot carcasses (*F*_5,87_ = 1.97, *P* = 0.09; Table 1), but ground squirrel mass was positively correlated with numbers of bullet fragments in carcasses (*F*_1,87_ = 8.63, *P* = 0.004). The number of fragments increased by 76%, on average (*β* = 0.004 ± 0.003 [95% confidence interval]; Fig 3A), across the range (54–521 g) of Belding’s ground squirrel carcass masses. After accounting for the mass of shot Belding’s ground squirrels we also found that the distribution of bullet fragments throughout carcasses differed by bullet type (*F*_5,74_ = 2.62, *P* = 0.03). However, after controlling for the overall alpha level in our Tukey’s pairwise tests we did not detect a significant difference in the distribution of bullet fragments between any of the bullet types (*P* >0.05). However, there was a general trend of the distribution of bullet fragments being greater in carcasses shot with .17 Super Mag bullets and random unknown bullet types (Table 1), whereas the distribution tended to be lower among all other bullet types (Table 1). Mass of ground squirrels did not influence the distribution of bullet fragments (*F*_1,74_ = 0.72, *P* = 0.39). Because we did not digest dermal material, estimates of bullet fragment numbers and mass in dermal material are derived strictly from the radiographic methods; across all carcasses we observed fragments in dermal tissues in 57% (17 of 30) of the carcasses, but the dermal tissue contained only 3.8% of the total number of fragments and 6.1% of mass of fragments associated with a ground squirrel carcass.

### Bullet fragments mass

**Fig 4:**
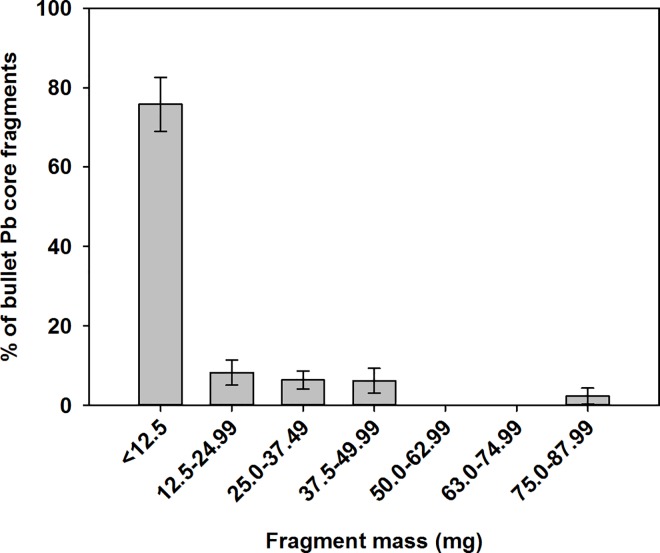
Distribution of the mass (mg) of carcass bullet fragments extracted from Belding’s ground squirrel carcasses.

**Fig 5:**
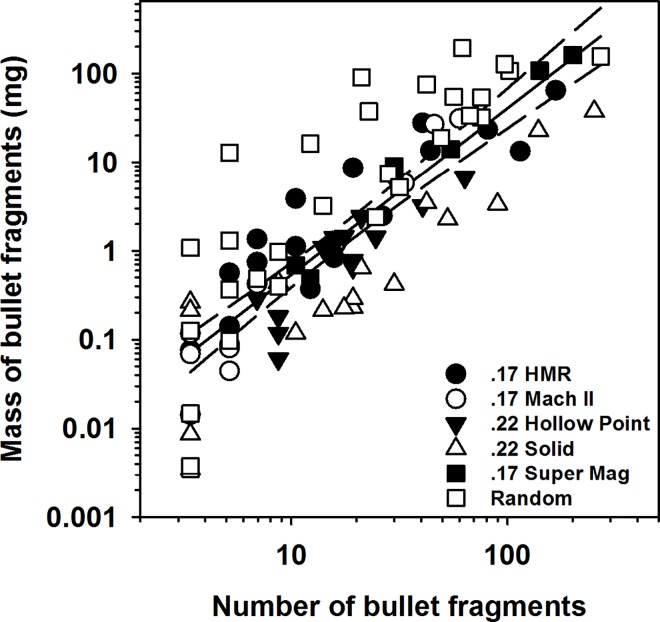
Correlation between the estimated mass (mg) of bullet fragments in ground squirrel carcass and estimated total fragment numbers from shot Belding’s ground squirrel carcasses.

Individual bullet fragment mass in digested ground squirrels ranged from 0.003 to 96.252 mg (*n* = 969) and the largest range within an individual squirrel was 0.008 to 96.252 mg. Most fragments recovered were relatively small; 76% of the fragments weighed less than 12.5 mg and had a mean surface area of 6.05 mm^2^ ± 2.61 SE (range = 0.04–56.01 mm^2^; Fig 4). Radiograph-derived bullet fragment area was highly correlated with the total mass of fragments recovered from digested ground squirrels (*F*_1,18_ = 8.81, *P* < 0.0001, *R*^2^ = 0.81; Fig 2B). Across all radiographed ground squirrels, estimated total fragment mass ranged from 0.003 to 195.35 mg per squirrel with an estimated surface area ranging from 0.01–63.90 mm^2^ (mean = 7.56 ± 1.38 SE). After accounting for mass of ground squirrel carcasses, we found that bullet type influenced the log mass of bullet fragments retained in carcasses (*F*_5,87_ = 5.71, *P* = 0.0001). Post hoc Tukey’s HSD tests found that ground squirrels shot with unknown and Super Mag .17 bullets retained more bullet fragment mass than .22 hollow point (*P* = 0.04) and .22 solid ammunition (*P* = 0.009) but similar amounts to .17 HMR and .17 Mach II (*P*>0.05; Table 1). Similarly, carcasses shot with random unknown bullet types retained more bullet fragment mass than .22 hollow point (*P* = 0.0005) and .22 solid ammunition (*P* = 0.004) but similar amounts to .17 HMR, .17 Mach II, and .17 Super Mag bullets (*P*>0.05; Table 1). Mass of ground squirrels was also positively correlated with mass of Pb fragments in carcasses (*F*_1,87_ = 9.91, *P* = 0.002). The total mass of Pb fragments increased on average by 56% (*β* = 0.009 ± 0.006; Fig 3B) across the range of Belding’s ground squirrel carcass masses. Lastly, we found that there was a strong correlation between the log estimated total number of bullet fragments and log estimated mass of bullet fragments (*F*_1,92_ = 14.27, *P* <0.0001, *R*^2^ = 0.69; Fig 5).

### Raptor nestling exposure

**Fig 6:**
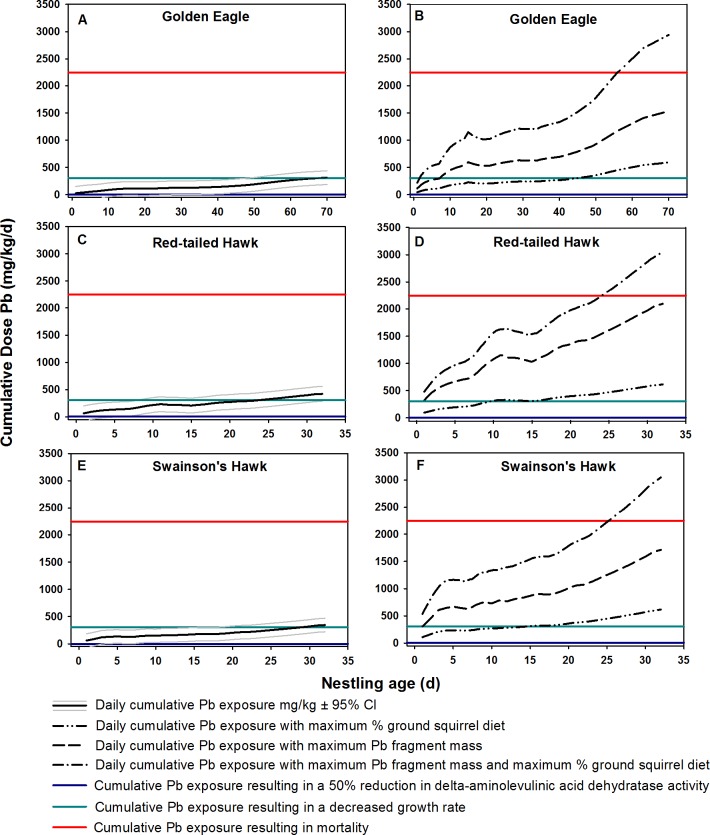
Daily cumulative Pb exposure (mg/kg body mass) for nestling golden eagles, red-tailed hawks, and Swainson’s hawks. Cumulative exposure thresholds for physiology, growth, and survival effects are shown as horizontal lines.

Under all model scenarios, the cumulative Pb dose for nestlings of all three raptor species exceeded the cumulative exposure threshold associated with a 50% reduction in delta-aminolevulinic acid dehydratase production for every day of the nestling stage (Fig 6A–6F). The growth effects threshold was surpassed for golden eagle nestlings at 52, 8, 45, and 2 days of age based on the field conditions, maximum ground squirrel diet, maximum Pb mass, and maximum ground squirrel diet and Pb mass models, respectively (Fig 6A and 6B). For golden eagle nestlings this would result in potential growth effects occurring for the final 26%, 89%, 36%, and 98% of the nestling stage under each modeling scenario, respectively. The growth effects threshold was surpassed for red-tailed hawk nestlings 18, 2, 10, and 1 days of age based on the field conditions, maximum ground squirrel diet, maximum Pb mass, and maximum ground squirrel diet and Pb mass models, respectively (Fig 6C and 6D). This would result in potential growth effects occurring for the final 44%, 94%, 59%, and 97% of the nestling stage under each respective scenario. The growth effects threshold was surpassed for Swainson’s hawk nestlings at 24, 2, 15, and 1 days of age based on the field conditions, maximum ground squirrel diet, maximum Pb mass, and maximum ground squirrel diet and Pb mass models respectively (Fig 6E and 6F). This would result in potential growth effects occurring for the final 25%, 94%, 53%, and 97% of the nestling stage under each respective scenario. Only under the maximum ground squirrel diet and maximum Pb mass model did nestling golden eagles, red-tailed hawks, and Swainson’s hawks exceed the survival threshold at 56, 25, and 27 days of age, respectively (Fig 6B, 6 and 6F). Across golden eagle, red-tailed hawk, and Swainson’s hawk nestlings this could result in survival effects occurring in the final 20%, 22%, and 16% of the nestling stage (Fig 6B, 6D and 6F).

## Discussion

Recreational ground squirrel shooting is a popular activity throughout the western United States and also serves as an important tool for managing ground squirrel populations, particularly in alfalfa fields [22,47]. Ground squirrels shot with Pb-based bullets may pose a risk to avian scavengers if Pb remains in the carcass and is available to scavenging birds. We found similar numbers of bullet fragments in ground squirrels across bullet types, but the total estimated fragment mass differed by ammunition type (e.g., Super Mag .17). One caveat to these findings is that all ground squirrels were shot under natural field conditions, thus factors that might influence fragmentation behavior of each bullet type such as shot placement, distance to target, and angle of entry were not standardized [34]. Although these factors may influence some of the fragmentation behavior of the bullets tested in this study, the results we present reflect the bullet’s behavior when shooting ground squirrels in the wild and thus reflect the potential Pb exposure to avian scavengers.

The distribution of bullet fragments in ground squirrel carcasses may play an important role in the likelihood of avian scavenger being exposed to Pb depending on the degree of fragment dispersal. Birds scavenging shot ground squirrel carcasses may be more likely to ingest Pb fragments if those fragments are distributed throughout the carcass as compared to just being clustered in a small area. We further expect that shot ground squirrel carcasses that are delivered to nestling birds with more dispersed fragments would increase the likelihood of Pb exposure. Although our post hoc models could not identify differences among bullet types as determined by the main effect of bullet type in our model, there was an apparent trend for carcasses shot with .17 Super Mag bullets to have fragments dispersed more than 2 times further than the other bullet types (Table 1). The .17 Super Mag bullets have much higher reported muzzle velocity (3000 fps/914 mps) relative to other known bullet types (mean = 1796 fps/547 mps), which likely resulted in this increased fragment dispersal. Higher muzzle velocities also contribute to increased bullet expansion and fragmentation upon impact [48].

The influence of target mass and bullet type has important implications for potential avian exposure to Pb fragments in ground squirrels and other related taxa. Similar to our findings, 87% of black-tailed prairie dog carcasses shot in Wyoming prairie dog colonies contained bullet fragments [21]. However, those prairie dog carcasses containing bullet fragments with nearly six times more Pb by mass relative to ground squirrels in the present study (228 vs 38.6 mg). We found that both the number and mass of retained fragments increased with increasing ground squirrels size, likely because the carcass had more tissue in which fragments become ensnared in larger ground squirrels [49]. Black-tailed prairie dog mass ranges between 650 and 1100g [50], which is 2.8–4.7 times heavier than the average Belding’s ground squirrel in the present study. Additionally, the prairie dogs were shot with expandable Pb bullets that were 2.1 times heavier (55 gr; 3554 mg; [21]) than those used on ground squirrels (mean = 26 gr; 1685 mg). Moreover, the muzzle velocity of ammunition used in the study of prairie dogs was relatively high (3250 fps (990 m s^-1^), increasing the potential for fragmentation and increased retention of fragments [48] in the larger black-tailed prairie dogs. However, it is unclear whether the increased fragment numbers and total mass in carcasses observed in Pauli and Buskirk [21] relative to ours resulted from higher velocity or heavier bullets or a combination of those factors.

Limitations to the accuracy and precision of radiograph images for estimating the number and mass of bullet fragments in ground squirrel carcasses can inhibit robust estimates of Pb exposure to avian scavengers. In both our study and the above study on black-tailed prairie dogs [21] where complete carcass digestion was utilized, radiographs underestimated the number of fragments. In particular, the slope of the line for the relationship between radiograph and carcass digestion fragment counts was substantially greater than one (*β* = 1.77), indicating that radiographic methods fail to detect a proportionally great number of fragments as the number of fragments in a carcass increases (Fig 2A). However, the fragment mass vs fragment area figure (Fig 2B) suggests that those fragments were very small, accounting for a very low proportion of residual bullet mass. Studies that only partially dissect and digest sections where visible fragments occurred may miss smaller fragments not captured by the radiograph. This is evidenced by a previous study that visually removed bullet fragments in shot black-tailed prairie dogs [51], where 40% of the carcasses were found to contain bullet fragments, in comparison to 80–87% in our study and Pauli and Buskirk’s [21] study respectively. Further, our results differed considerably from the above study that visually removed bullet fragments [51] and one additional study that examined bullet fragments in shot Richardson’s ground squirrel carcasses; see [20]. Our digested ground squirrels either had more Pb mass [20], or less mass but more fragments [51], than those two previous studies. However, in both of those studies ground squirrels were not completely digested and their results may not accurately reflect either the total number or mass of Pb fragments in the carcasses. Although the overall mass of missed fragments may be small, the potential for those smaller fragments to be absorbed into the blood stream is greater given their larger surface area to mass ratio [33]. We caution that future studies utilizing radiographs to identify the number of bullet fragments in shot carcasses should consider first digesting a subset of carcasses to estimate the relationship between radiographed estimates with actual estimates of fragment numbers or mass. Similarly, studies that have previously examined the number of Pb-bullet fragments retained in gut piles or carcass of deer or surrogate deer species (e.g., sheep) using only radiograph images; see [52–55] may have underestimated the true number of fragments retained and thus potential risk to avian scavengers, although validating those estimates using digestion procedures may be logistically infeasible.

Across bullet types, less than 1% of the original bullet mass remained in ground squirrel carcasses as fragments. This differs considerably other estimates where over 7% of the original bullet mass remained in shot ground squirrel carcasses [21]. As discussed above, these differences are likely driven by the mass of the species of ground squirrel being shot and the ammunition type used. Yet some carcasses retained enough Pb to be potentially lethal to nestling birds if ingested in a single meal. In fact, 7% (9 of 127) of carcasses in our study exceeded 60 mg of Pb, a level of cumulative exposure that has resulted in 40% mortality in nestling American kestrels (*Falco sparverius*) after six days of dosing [45]. Thus, we conclude there is an elevated likelihood that mortality could result from a nestling consuming a single shot ground squirrel delivered by an adult avian scavenger in a single meal.

Estimating potential risk to nestlings from this form of Pb exposure is complicated by a number of ecological and toxicological factors such as diet, changing energetic requirements of growing nestlings, Pb assimilation rates, and differences in Pb toxicity among species. We used literature-derived estimates of effects of Pb exposure on nestling physiology, growth and survival to model potential cumulative Pb exposure throughout the nestling stage for golden eagles, red-tailed hawks, and Swainson’s hawks. Under all models, we found that all species exceeded the 50% reduction in delta-aminolevulinic acid dehydratase production threshold for the entire nestling stage. It is unclear what, if any, potential long term effects could occur from such a substantial reduction in delta-aminolevulinic acid dehydratase production; however, nestlings have been able to recover after reduction to less than 5% of normal without severe clinical hematological effects [56]. However, recent experimental Pb pellet dosing studies on red-legged partridges (*Alectoris rufa*) have shown that even low one-time doses of 109 mg resulted in substantial suppression of delta-aminolevulinic acid dehydratase production, and activity levels were only 34% of normal after 21 days [57]. Regardless, recovery only occurs when Pb exposure ceases. Despite this, a 50% reduction in delta-aminolevulinic acid dehydratase production could result in anemic conditions in nestlings [45]. To our knowledge, no studies have examined the response of delta-aminolevulinic acid dehydratase production in any of these species’ nestlings to Pb exposure in the wild.

Reduced growth rates of nestlings can also occur in association with Pb exposure, although generally only at high doses [45,58]. Under the field conditions model, the growth effects threshold was not exceeded until nestlings were between 18 to 52 days of age or 73–87% of the time through the nestling stage for golden eagles, red-tailed hawks, and Swainson’s hawks. At this stage, nestlings have achieved 89–100% of their growth [39–42], thus it is unlikely that a significant reduction in growth would be expected. However, under the maximum ground squirrel diet, maximum Pb mass, and maximum ground squirrel diet and maximum Pb mass models, the growth effect threshold was exceeded when nestlings ranged from 1 to 45 days of age or 63–100% of the time through the nestling stage across all species. Among the most serious potential effects of reduced growth associated with Pb exposure is a reduction in brain weight. Lead exposure in nestling American kestrels [45], European starlings (*Sturnus vulgaris*; [59]), and mallard ducklings (*Anas platyrhynchos*; [60]) has been linked to reduced brain weights. Although unsubstantiated in birds, decreased mammalian brain weights associated with Pb exposure resulted in decreased cognitive function [61,62].

Experimental Pb exposure effects on raptor nestling survival have only been found to occur under relatively high Pb doses (e.g., 625 mg/kg body mass; [45]). Correspondingly, we estimated that raptor nestlings would exceed the survival threshold in only the maximum ground squirrel diet and maximum Pb mass model when nestlings reached 25 to 56 days of age or the final 21–25% of the nestling stage. This particular model was intended to examine Pb exposure to nestling raptors under a potential worst-case scenario for ground squirrel shooting. However, we note that all of our models were developed only to look at Pb exposure associated with shot ground squirrels, and as such do not incorporate other potential sources of Pb from the landscape where these birds nest. For instance, coyotes (*Canis latrans*) and a variety of rabbit species (Leporidae) are also shot as pests during the nesting season; these carcasses also could contain Pb fragments and would increase the overall Pb burden in nestling raptors [63,64]. Additionally, chukar (*Alectoris chukar*) are common in the diets of golden eagles and recent research has shown that nearly 10% of chukars’ gizzards contained Pb-based pellets [65]. As an additional caveat to our nestling exposure modeling, we note that we utilized estimates of Pb only from ground squirrels shot in our study areas. In this study area, shooters typically used .17 or .22 caliber rifles and the mass of Pb retained was small relative to other studies; see [21]. In other regions where ground squirrel shooting is common, shooters often use larger calibers (e.g., .223; [21]). If Belding’s ground squirrels were shot with these larger calibers, we expect that carcasses would retain more Pb mass than observed in our study. Without knowing what proportion of those larger caliber bullets would be retained in a shot Belding’s ground squirrel it is difficult to determine exactly how much of an increase in Pb exposure and effects risk might occur.

Despite these uncertainties, these findings illustrate the importance of more accurate estimates of raptor dietary reliance (and other avian scavengers) on shot carcasses, and strengthening these estimates would improve our knowledge of the risk of Pb-laced ground squirrels to nestling physiology, growth, and survival. For some species that rely heavily on already dead prey and in particular prey that have been shot (e.g. common ravens; [14,66]), we expect that the likelihood of Pb exposure effects in those nestlings would be even higher than for the raptor species modeled in our study. Additionally, the effects of Pb exposure are not limited to those few chosen in our modeling efforts. Recent research has shown that Pb can be maternally transferred to avian embryos resulting in inhibited blood delta-aminolevulinic acid dehydratase activity, negative immune responses, and increased oxidative stress levels [57]. Nestling exposure to Pb in their diet can also result in negative effects on immune parameters [67], lower hemoglobin levels [68], less vigorous food-acquisition behaviors and poor coordination [2], among other effects. Furthermore, adult facultative and obligate avian scavengers also scavenge dead ground squirrels [25–27] and would be exposed to lead during the breeding season. The exact effects of lead exposure associated with ground squirrel shooting in adult avian scavengers is unknown, however, lead exposure associated with other forms of hunting or recreational shooting can result in physiological effects [1,3,12] to outright mortality [64]. For long-lived raptors, physiological and survival effects of lead exposure on adults are likely more critical to demography than effects that may influence productivity measures [69,70].

### Conservation and management implications

Differences in the bullet fragment mass retained in carcasses among ammunition types may be a valuable factor for supporting management decisions. We found that ground squirrels shot with .17 Super Mag bullets retained over 28 times as much bullet fragment mass than either .22 solid or .22 hollow point bullets. Outreach and education efforts that communicate how bullet type influences potential scavenger exposure may be a useful approach for resource management. Reducing the number of carcasses on the landscape that contain bullet fragments may be another useful tool for reducing Pb exposure in avian scavengers [20,64,71]. However, recent modeling efforts examining different mitigation scenarios associated with big game hunting (e.g., gut pile removal vs non-lead ammunition) to reduce Pb exposure in golden eagles consistently showed that the use of non-Pb ammunition is likely more effective than removing gut piles in reducing eagle mortality [72]. Further, these effects became even stronger when densities of gut piles on the landscape increased [72]. It is unclear whether these results are directly transferrable to the case of ground squirrel shooting, where hundreds of shot carcasses may be left on a field in the presence of even larger large numbers of live ground squirrels.
